# The first reported cases of elephant endotheliotropic herpesvirus infectious haemorrhagic disease in Malaysia: case report

**DOI:** 10.1186/s12985-021-01694-x

**Published:** 2021-11-24

**Authors:** Mei-Ho Lee, Senthilvel K. S. S. Nathan, Laura Benedict, Pakeeyaraj Nagalingam, Erin Latimer, Tom Hughes, Diana Ramirez, Jum Rafiah Abd Sukor

**Affiliations:** 1 Conservation Medicine, 47000 Sungai Buloh, Selangor Malaysia; 2 Sabah Wildlife Department, 88100 Kota Kinabalu, Sabah Malaysia; 3 EcoHealth Alliance, New York, NY 10018 USA; 4 National Elephant Herpesvirus Laboratory, Wildlife Health Sciences Department, Smithsonian’s National Zoo, Washington, DC 20008 USA; 5 Wildlife Rescue Unit, Sabah Wildlife Department, 88100 Kota Kinabalu, Sabah Malaysia

**Keywords:** Bornean elephant, Elephant endotheliotropic herpesvirus, Sabah, Malaysia

## Abstract

**Background:**

Elephant endotheliotropic herpesvirus haemorrhagic disease (EEHV HD) is the leading cause of death in captive Asian elephant calves in Asia, North America, and Europe with a mortality rate of ~ 65% in calves that are under human care. Although EEHV HD was first found in elephant camps, more recently it was identified in wild populations which poses a greater threat to the elephant population. Deaths due to EEHV HD have been seen in wild elephants, but the in-situ prevalence and mortality rate is unknown. We report the first EEHV HD cases in Malaysia from 3 wild born endangered Bornean elephant calves from Sabah with known typical clinical signs.

**Case presentation:**

The first calf died within 24 h of the onset of clinical signs; the second calf died within 12 h of the onset of clinical signs. The third calf succumbed within 72 h. Necropsies revealed that all 3 calves had similar presentations of EEHV HD but in the third calf with less severity. We conducted conventional polymerase chain reaction (cPCR) assays and found EEHV DNA at all 7 loci in the 3 calves; it was identified as EEHV1A, the virus type that has been found in most other reported cases.

**Conclusion:**

Typical EEHV HD clinical signs and the molecular confirmation of EEHV by cPCR and sequencing point to EEHV as the cause of death. Further genetic investigation of the strain is in progress.

## Background

Elephant endotheliotropic herpesvirus (EEHV) was the first and so far, only described herpesvirus causing critical disease in elephants with visible morphological changes [1] and resulted in an 85% fatality rate in infected individuals [2, 3]. Following a novel EEHV report [2], more than 100 similar cases have been identified across the globe. Captive born juvenile Asian elephants (*Elephas maximus*) were affected in the majority of the confirmed cases [4, 5]. Over the past 20 years, this disease has had a mortality rate of 65% in young Asian elephants between 3 months to 15 years of age in Europe and North America in human care [3]. Furthermore, EEHV haemorrhagic cases are increasingly reported in the endemic wild and captive populations in Asia [3, 6–9].

Most EEHV cases linked with systemic endotheliolytic disease affecting Asian elephants belong to the EEHV1 group (EEHV1A and EEHV1B), although there has been several cases of morbidity and mortality due to EEHV4 and EEHV5 in Asian elephants [10–14]. Historically there had been two deaths due to EEHV2 in African elephants (*Loxodonta africana*) and a death due to EEHV6, [4, 15] as well as a survivor of EEHV3B [16] and EEHV6 [13]. Since 2019, there have been three deaths, five survivors of EEHV HD, and five cases of barely clinical/subclinical EEHV, all due to either EEHV3A or EEHV3B, as well as a death from EEHV2 and what seems to have been a well-controlled viraemia from EEHV6 [17, Latimer E, personal communication]. Though the transmission mechanisms of EEHV have not been fully understood, viral DNA is found in trunk secretions and other bodily fluids and is believed to be involved in transmission [18].

EEHV-infected elephants display an acute onset of lethargy, generalised oedema of head and limbs, oral ulceration, cyanosis of the tongue, tachycardia, and death after a period of 1–7 days. Lymphopenia and thrombocytopenia are commonly seen in blood evaluations. Increasingly, there are reports of elephants surviving EEHV HD with treatment including antivirals, fluids, platelets, and immunostimulants, among other therapies [4, 17, 19–23]. Pericardial effusion, intestinal haemorrhage, and mucosal ulcerations are common necropsy findings. Target tissues for EEHV include heart, tongue, liver, and large intestine. Histological examinations usually reveal microhaemorrhages, oedema and inflammation in these organs. Lesions can be accompanied by intranuclear herpesvirus inclusions in capillary endothelial cells [1].

There is no prior report published of EEHV infection in Malaysia or from the Bornean elephant—a subspecies of Asian elephant on the island of Borneo, mainly in Sabah, a state in East Malaysia. Bornean elephants, *Elephas maximus borneensis,* are one of the four distinct subspecies of the Asian elephants. They are classified as endangered according to the International Union for Conservation of Nature (IUCN) Red list of threatened species due to habitat loss. More recently, the species is also threatened from illegal hunting, poisoning and attacks by those trying to protect crops. Sabah has an ex-situ population of 24 elephants in human care at the Sepilok Orangutan Rehabilitation Centre, (Sandakan, SORC), Borneo Elephant Sanctuary (Kinabatangan) and Lok Kawi Wildlife Park (Kota Kinabalu). The state’s wild population is estimated at 2000 individuals [24].

There are two hypotheses regarding the origins of these elephants—they were introduced to Borneo either from Sulu [25] or Sumatera and Peninsular Malaysia or were indigenous to Borneo but derived from a Sundaic stock [26]. Genetic studies of mitochondrial DNA divergence supports the latter hypothesis [26, 27]. In the wild, they can be found at the northern and north-eastern parts of Borneo in the state of Sabah in Malaysia and the upper Sembakung River in Kalimantan, Indonesia [28]. With the many rampant threats including EEHV HD, low population numbers and the uniqueness of the species in terms of its habitat range and genetic makeup, the conservation and the health of this species is tremendously important to ensure the survival of this iconic species in the wild and in human care.

## Case presentation

**Fig. 1:**
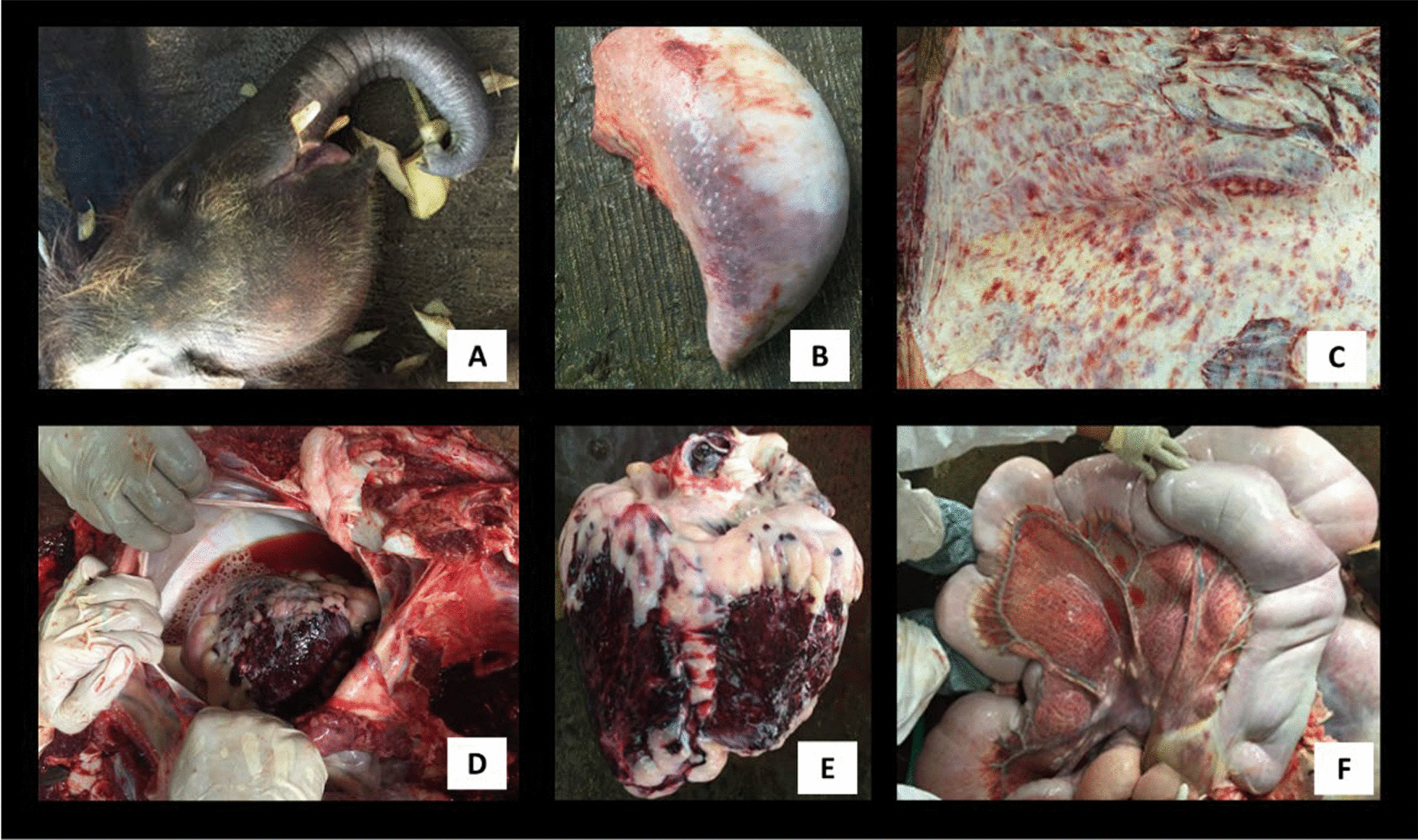
Necropsy findings of EEHV. **A** Marked facial and trunk oedema in the first case with tongue cyanosis. **B** Tongue discolouration due to internal haemorrhaging in the second case (also observed in the first case). **C** Diffuse subcutaneous petechiation observed in all 3 cases. **D** Pericardial effusion from the first case but present in all three cases. **E** Heart of the first case, severe cardiac haemorrhage. **F** Generalised haemorrhage of gastrointestinal tract observed in all 3 cases

The first occurrence of EEHV infection at SORC occurred in a 24-month-old *E. m. borneensis* calf (Table 1). Symptomatic treatment (prednisolone 1 mg/kg, papase, oxytetracycline 20 mg/kg, intravenous fluid therapy) was administered. The calf died within 24 h of the onset of clinical signs. Over 1 L of serosanguinous epicardial fluid (Fig. 1D) was retrieved from the pericardial sac. The calf had facial swelling (Fig. 1A). Over 6–12 h the swelling increased, and discolouration of the tongue apex was observed (Fig. 1B). The necropsy revealed prominent facial and truncal oedema with cyanotic swollen tongue. Generalised subcutaneous petechiation (Fig. 1C) with moderate subcutaneous oedema was observed. Severe haemorrhaging was evident in the heart extending from the epicardium through the myocardium and papillary musculatures (Fig. 1E). Generalised haemorrhage and oedema along the gastrointestinal tract including the mesentery was observed (Fig. 1F), and the liver was enlarged with mild petechiation.

**Table 1:** Details of the *E. m. borneensis* tested and their PCR results

Animal	Date and place of rescue	Sex	Age and weight during the outbreak	Date of initial disease signs	1st collection (11 May 2016)	2nd collection (25 May 2016)	PCR results with sequencing confirmation
Jimbo (First case)	February 2014; Beluran, Sabah	Male	24 months; 362 kg	9th May 2016	Not collected	Not collected	+*
Vinodh (Second case)	November 2014; Telupid, Sabah	Male	24 months; 442 kg	17th May 2016	WB, S	WB, S	+
Tuntan (Third case)	February 2014; Sukau, Sabah	Male	36 months; 478 kg	20th May 2016	WB, S	S	+
Danum	December 2015; Lahad Datu, Sabah	Male	24 months; 289 kg	N/A	WB, S	S	−
Budak	February 2016, Kinabatangan, Sabah	Male	5 months; 97.5 kg	N/A	WB, S	S	−
Tunku	February 2016, Kinabatangan, Sabah	Male	5 months; 118 kg	N/A	WB, S	S	−
Adun	April 2015; Telupid, Sabah	Male	12 months; 247 kg	N/A	WB, S	S	+
Dumpas	August 2015; Tawau, Sabah	Male	21 months; 372 kg	N/A	WB, S	S	−

WB, whole blood; S, serum; +, EEHV detected; −, EEHV not detected; N/A, not applicable

*PCR confirmation from organs, not from WB using subsequent PCR protocols only

Following the first calf’s death, all other elephants at the SORC were administered acyclovir 19 mg/kg, oxytetracycline 20 mg/kg, vitamin C and virgin coconut oil supplements. The second EEHV case occurred 8 days later in another 24-month-old calf (Table 1). Intravenous fluid therapy was administered immediately, but the calf died less than 12 h later. The third death occurred 3 days later in a 36-month-old calf (Table 1). Treatment as described above was given. The calf regained appetite for milk and solid food the next day following intensive fluid therapy (rectal and intravenous). Nevertheless, on the next morning, the calf stopped eating and died several hours later.

The necropsy of the second dead calf revealed similar findings to the first one. The necropsy of the third calf showed similar findings to the first two calves but with less severity. Dullness, inappetence and lethargy were observed in all cases. While tongue cyanosis and swelling, and facial and trunk oedema were only observed in the first two calves. Vesicles and oral ulceration were not observed in all three calves.

Organ samples were collected from all three dead calves for *Escherichia coli* culture and histopathological examination. Blood samples from the last two dead calves and from five apparently healthy elephants from the SORC (Table 1) were stored and tested at the Wildlife Health, Genetic and Forensic Laboratory (WHGFL), Kota Kinabalu, Sabah, a certified biosafety level 2 laboratory, at the request of the Sabah Wildlife Department and with Sabah Biodiversity Centre approval (SaBC License Number: JKM/MBS.1000-2/2JLD.5). DNA was extracted from these samples using the QIAamp Blood and Tissue Mini Kit (Qiagen, Hilden, Germany). For immediate diagnosis, conventional PCR amplifications were conducted on the extracted DNA for herpesviruses using consensus [29, 30] and EEHV PCRs [4, 13] (Table 2). For further genetic investigations on the variabilities on these loci, all blood and organ samples were rescreened at WHGFL with PCRs on conserved gene loci—U60(TERex3), U71(gM), and U77(HEL) and on hypervariable gene loci—U48.5(TK), U48(gH-TK), U51(vGPCR1), and E54(vOX2-1) [Hayward G, unpublished data, 31] (Table 3). Since the serum and whole blood from the first dead calf were unavailable, initial consensus and EEHV PCRs were not performed for this animal. Instead, the organ samples were used for the subsequent EEHV PCRs.

**Table 2:** Initial diagnosis PCR protocols and primers used

Target gene	Primer sequences (5′-3′)	Product length (bp)	References
Terminase	Round 1: TS-TERM_707s: TTGTGGACGAGRSIMAYTTYAT TS-TERM_707as: ACAGCCACGCCNGTICCIGAIGC Round 2: TS-TERM_708s: GCAAGATCATNTTYRTITCITC TS-TERM_708as: TGTTGGTCGTRWAIGCIGGRT	Round 1: 519 Round 2: 419	[29]
Polymerase	Round 1: DFA: GAYTTYGCNAGYYTNTAYCC KG1: GTCTTGCTCACCAGNTCNACNCCYTT ILK: TCCTGGACAAGCAGCARNYSGCNMTNAA KG1: GTCTTGCTCACCAGNTCNACNCCYTT Round 2: TGV: TGTAACTCGGTGTAYGGNTTYACNGGNGT IYG: CACAGAGTCCGTRTCNCCRTADAT	Round 1: DFA/KGI: ~ 750 ILK/KGI: ~ 480 Round 2: 215–315	[30]
EEHV DNA Polymerase- Specific EEHV 3/4	6719: CGTTGAAGGTGTCGCAGAT 7400: CAGCATCATCCAGGCCTACAAC 6720: ATCCTGGCGCAGCTGCTGAC 6721: CTCACCTGCAACGCCGTCTA	Round 1: 6719/7400: 390 Round 2: 6719/6720: 270 Round 3: 6719/6721: 150	[13]
EEHV DNA Polymerase- PAN EEHV and specific EEHV 6	6710: ACAAACACGCTGTCRGTRTCYCCRTA 6711: GTATTTGATTTYGCNAGYYTGTAYCC 6712: TGYAAYGCCGTNTAYGGATTYACCGG 7584: CATCGATTTTGAACTTCTCATGGTC	Round 1: 6710/6711: 500 Round 2A: 6710/6712: 250 Round 2B: 6711/7584: 500 Round 3A: 6712/7584: 250	[13]
EEHV DNA Terminase	B1 LGH2425: ACAGCCACGCCNGTNCCNGANGC A2 LGH2428: TTGTGGACGAGRSNMAYTTYAT A3 LGH2426: GCAAGATCATNTTYRTNTCNTC B2 LGH2427: TGTTGGTCGTRWANGCNGGRTC A3SEQ: CCCCATCTGAGCAAGATCAT B2SEQ: GGCTGACAAATGTTGGTCGT	Round 1: B1/A2: 575 Round 2: A3/B2: 415 A3SEQ/B2SEQ: 360	[4]

**Table 3:** Subsequent PCR protocols and primers used

Genes/loci	Primer sequences (5′-3′)	Product length (bp)	Accession number
U60(TERex3)	LGH6640: AAATGTTCTATTCCGTATAC LGH6672: CATGTTGTGCAGGCACTCTTC LGH6671: GTTTGTAGTAAATGCCGGATC LGH6672: CATGTTGTGCAGGCACTCTTC	Round 1: 6640/6672: 850 Round 2: 6671/6672: 780	JX011056-62
U71(gM)	LGH6749: CTATGGGATCCGAACTTTC LGH6752: CTACATGCCCATGCAGATAGG LGH6750: CTTTCTAAGGGGGTTTGTTGC LGH6752: CTACATGCCCATGCAGATAGG	Round 1: 6749/6752: 730 Round 2: 6750/6752: 710	JX011063-71
U77(HEL)	LGH6743: GCAAGGTRGAACGTATCGTCG LGH6742: CACAGMGCGTTGTAGAACC LGH6743: GCAAGGTRGAACGTATCGTCG LGH6742: CACAGMGCGTTGTAGAACC	Round 1: 6732/6742: 680 Round 2: 6743/6742: 680	JX011072-79
U48.5(TK)	LGH6764: GCACGRTACCACGTACTC LGH7968: GCGGCAACGAAGTTCACAGGCATYATGG LGH7968: GCGGCAACGAAGTTCACAGGCATYATGG LGH7970: TGCMGCYTGAAGGCTGTTTATATACT	Round 1: 6764/7968: 750 Round 2: 7968/7970: 640	MN864103.1
U48(gH-TK)	LGH7981: CTRCATTKMCCAAAGTATGGAAGTA LGH7985: GGTAGGTTCACCTACATGGAACTTC LGH7982: CRTYTATATCATCAAARACYTCACA LGH7985: GGTAGGTTCACCTACATGGAACTTC	Round 1: 7981/7985: 1080 Round 2: 7982/7985: 1040	JX011039-46
U51 (vGPCR1)	LGH7506: GATTGTGAACGCTGTATGCT LGH4963B: GACTTTCTTCGTAGCCCTCGTCTT LGH7506: GATTGTGAACGCTGTATGCT LGH5200A: CGTGATACGCTTCAAAACATACA	Round 1: 7506/4963B: 910 Round 2: 7506/5200A: 750	JX011047-55
E54(vOX2-1)	LGH8471: ATGCTTCAGAGAAAGTACAGGTAC LGH8472: GTGTTGCCGCCACGATGCTTCTACG LGH8471: ATGCTTCAGAGAAAGTACAGGTAC LGH8506: CTA CGC CAC TTG TAA CAG AAT ATC ACG	Round 1: 8471/8472: 910 Round 2: 8471/8506: 890	MF464882-899

**Fig. 2:**
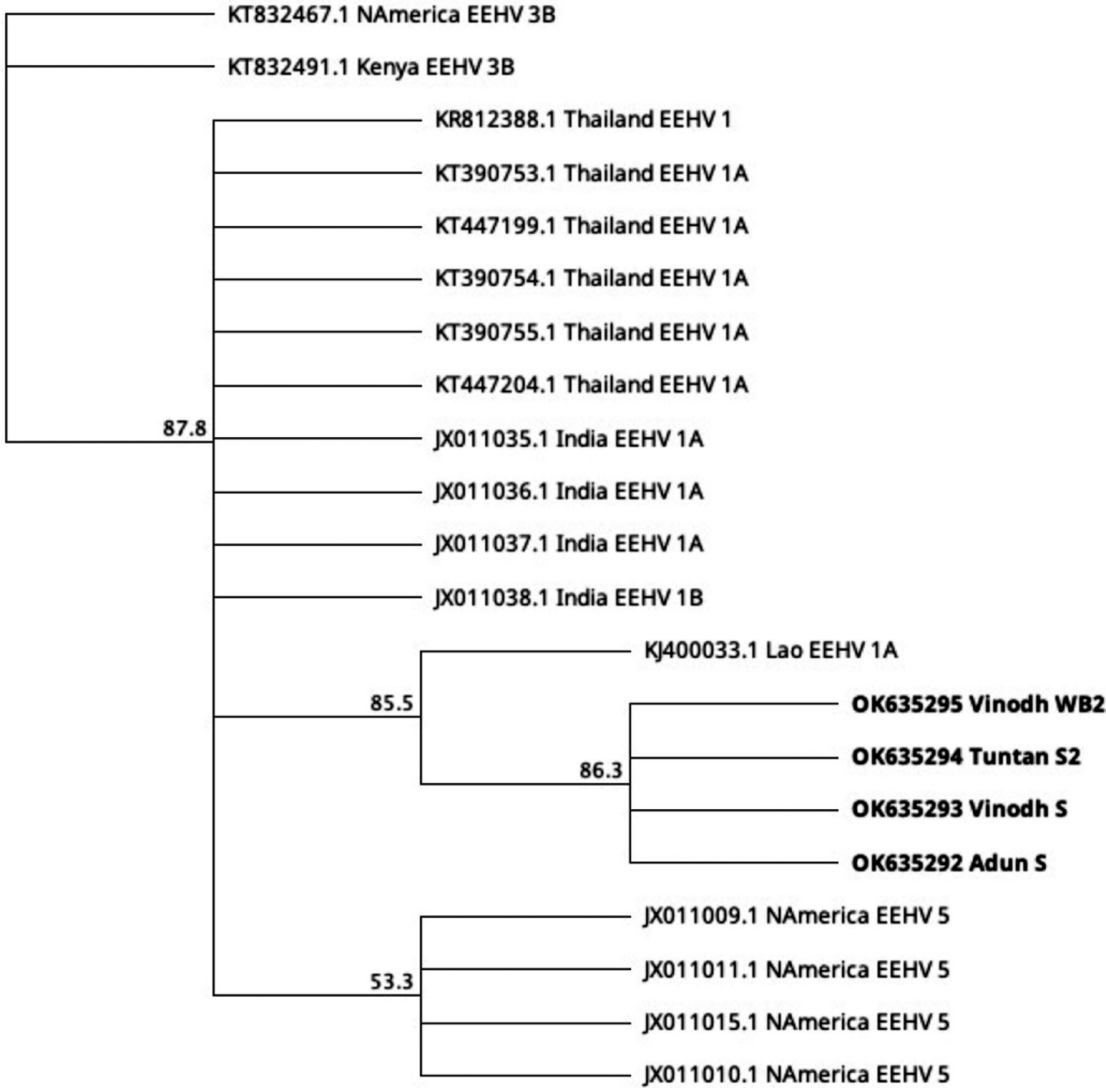
Phylogenetic tree of EEHV DNA polymerase of 2 dead and 1 live calf from the SORC (Accession no. OK635292, OK635293, OK635294, OK635295) with those from North America (Accession no. JX011009.1, JX011010.1, JX011011.1, JX011015.1, KT832467.1), Kenya (Accession no. KT832491.1), Thailand (Accession no. KT390753.1, KT390754.1, KT390755.1, KT447199.1, KT447204.1, KR812388.1), India (Accession no. JX011035.1, JX011036.1, JX011037.1, JX011038.1) and Lao (Accession no. KJ400033.1). *WB2* whole blood from second collection, *S* serum, *S2* serum from second collection

Correct sized PCR products on electrophoresis agarose were excised and purified using the QIAquick Gel Extraction Kit (Qiagen, Hilden, Germany). Purified products were sequenced using their corresponding specific primers. The nucleotide sequences were analysed with the Geneious 10.1.3 software (Auckland, New Zealand) and compared to sequences in GenBank for homology analysis using the Basic Local Alignment Search Tool (BLAST; National Center for Biotechnology Information). Cleaned sequences from the initial EEHV PCR were aligned with 17 published EEHV DNA polymerase sequences using the Geneious Prime 2021.1.1 software (Auckland, New Zealand) Geneious Alignment (Alignment type: Global alignment with free end gaps; Cost matrix: Identity, Table 4). Aligned sequences were used to build a phylogenetic tree using the Geneious Prime 2021.1.1 software (Auckland, New Zealand) Geneious Tree Builder, with the Jukes-Cantor genetic distance model and 1000 bootstrap replicates (Fig. 2).

**Table 4:** Cleaned sequences of the initial PCR of pan EEHV DNA polymerase, Round 2A using 6710/6712 [13]

Accession number and sample name	Sequence (length in bp)
OK635292 Adun S	TTGAATCCTATTACTGTCTACCGGGCAGTCAACTAGTTCGGGAGCTATTTGCGTTAAGAACGTCCAATCGTTAAATCTGTCGCAAATATATTGGTTGGTAACGGCCAGCAGTTGTCTGCCCTGCGCCGTGACCGATTCAGCTATGGCCAGACAGGGAAACATCCCTTTCGACACACCGGTGAATCCATACACGGCATTGCAAA (203)
OK635293 Vinodh S	TCGATTTTGATCTATTACTGTCTACCGGGCAGTCAACTAGTTCGGGAGCTATTTGCGTTAAGAACGTCCAATCGTTAAATCTGTCGCAAATATATTGTTTGGTAACGGCCAGCAGTTGTCTGCCCTGCGCCGTGACCGATTCAGCTATGGCCAGACAGGGAAACATCCCTTTCGACACACCGGTGAATCCATACACGGCATTGCAAA (207)
OK635294 Tuntan S2	CATCGATTTTGATCTATTACTGTCTACCGGGCAGTCAACTAGTTCGGGAGCTATTTGCGTTAAGAACGTCCAATCGTTAAATCTGTCGCAAATATATTGTTTGGTAACGGCCAGCAGTTGTCTGCCCTGCGCCGTGACCGATTCAGCTATGGCCAGACAGGGAAACATCCCTTTCGACACACCGGTGAATCCATACACGGCATTGCAA (208)
OK635295 Vinodh WB2	TCGATTTTGATCTATTACTGTCTACCGGGCAGTCAACTAGTTCGGGAGCTATTTGCGTTAAGAACGTCCAATCGTTAAATCTGTCGCAAATATATTGTTTGGTAACGGCCAGCAGTTGTCTGCCCTGCGCCGTGACCGATTCAGCTATGGCCAGACAGGGAAACATCCCTTTCGACACACCGGTGAATCCATACACGGCATTGCAAG (207)

*WB2* Whole blood from second collection, *S* serum, *S2* serum from second collection

Histopathological examination showed that inclusion bodies were insignificant in the organs of the three calves, but the histopathological lesions were suggestive of enteritis in the first calf and viral infection in the second and third. Using the consensus terminase and polymerase herpesvirus, the polymerase specific EEHV 3/4 and the EEHV terminase PCR assays, herpesvirus was not detected in all tested samples. Of the seven individuals from the SORC screened using the polymerase pan EEHV PCR assays, three individuals (second and third dead calves and one of the five healthy animals) were sequence-confirmed for EEHV, with 91–93% identical sequences to EEHV 1A and 1B (North American isolates) and elephantid herpesvirus 1 DNA polymerase. Phylogenetic analysis showed that there was 100% sequence agreement within the three calves with the initial EEHV PCR detection and they were more related to EEHV 1A from Lao (Accession no. KJ400033.1) than the other sequences used for the comparison. The EEHV detection for the first dead calf was confirmed with sequences from the follow-up PCRs. The detailed analysis of the results from the follow-up genetic investigations will be presented in a subsequent manuscript.

## Discussion and conclusions

These are the first reported cases anywhere of EEHV HD in Bornean elephants (*Elephas maximus borneensis)*. However, the lack of previous reports in Malaysia does not necessarily indicate a low EEHV HD morbidity. Initially, EEHV was hypothesised to be a novel virus for Asian elephants spread via exposure to African elephants in captivity [2]. However, in 2012, Asian elephants were shown to be the host with detection in wild Indian elephants [3] as well as other subsequent detections of EEHV in wild and camp elephants in Asia [9, 31]. Our cases support the latter, as the affected animals had no prior contact with African elephants.

Shedding by herd members could be the source of infection in vulnerable calves; the source of the SORC outbreak is not known. The EEHV may have been shed by the two youngest calves, aged less than 12 months old rescued four months prior to the outbreak, that may have still been protected by maternal antibodies. The calves that died were older than 24 months; none of the younger calves exhibited any clinical signs, although EEHV was detected in one of them. This is consistent with other fatal cases in individuals above 24 months old [4, 10], with maternal antibodies generally waning by 24 months old, explaining the higher fatality in slightly older calves [32].

Most fatal EEHV HD cases are of juvenile Asian elephants, ranging from 12 to 84 months old and usually dying within 24 h of the first detectable clinical signs [2, 10]. The course of the disease in the first two calves was peracute, both dying in less than 24 h from the onset of clinical signs, the third calf died after 72 h. Viraemia can occur several days prior to the onset of clinical signs and by this time severe vascular lesions would have developed [10]. Nevertheless, keepers should still be trained to identify the signs of the disease. Immediate administration of antiviral drugs might prevent further vascular damage, but its efficacy remains unclear and needs more investigation [23, 33]. A symptomatic treatment regime, including intensive fluid therapy, anti-inflammatories, parenteral antibiotics, and vitamins, remains crucial to reduce or stop the effects of initial vascular damages [33]. The efficacy of lauric acid found in the virgin coconut oil given to the elephants for its supposedly antiviral properties [34] in the treatment of EEHV HD is inconclusive because we were not able to distinguish between the effects from the drug treatments and from the supplements. At the time of the outbreak, there was limited information available on successful treatments, new treatment guidelines published subsequently [19–23] provide useful guidance for future outbreaks.

Since this was the first time we found EEHV in our elephants, it is important to determine the prevalence, patterns of shedding and the duration of the disease. In addition, early detection by PCR is vital for preclinical viraemia detection; routine screening serves as an early warning system to better prepare elephant centres for managing imminent EEHV outbreaks. PCR monitoring after an outbreak event is also crucial. These approaches can help us to understand EEHV HD to reduce morbidity and mortality in wild and captive elephants that are already endangered through habitat loss and increasing conflict with humans.

## Data Availability

The datasets used and/or analysed during the current study are available from the corresponding author on reasonable request.

## Abbreviations

EEHV HD: Elephant endotheliotropic herpesvirus haemorrhagic disease
cPCR: Conventional polymerase chain reaction
IUCN: International Union for Conservation of Nature
SORC: Sepilok Orangutan Rehabilitation Centre, Sandakan, Sabah
WHGFL: Wildlife Health, Genetic and Forensic Laboratory, Kota Kinabalu, Sabah
SaBC: Sabah Biodiversity Center
BLAST: Basic local alignment search tool
